# Prognostic accuracy of visual lung damage computed tomography score for mortality prediction in patients with COVID-19 pneumonia: a systematic review and meta-analysis

**DOI:** 10.1186/s43055-022-00741-z

**Published:** 2022-03-10

**Authors:** Seyed Salman Zakariaee, Negar Naderi, Danial Rezaee

**Affiliations:** 1 Department of Medical Physics, Faculty of Paramedical Sciences, Ilam University of Medical Sciences, Ilam, Iran; 2 Department of Midwifery, Faculty of Nursing and Midwifery, Ilam University of Medical Sciences, Ilam, Iran

**Keywords:** COVID-19, Computed tomography, Mortality, Sensitivity, Specificity

## Abstract

**Background:**

Chest computed tomography (CT) findings provide great added value in characterizing the extent of disease and severity of pulmonary involvements. Chest CT severity score (CT-SS) could be considered as an appropriate prognostic factor for mortality prediction in patients with COVID-19 pneumonia. In this study, we performed a meta-analysis evaluating the prognostic accuracy of CT-SS for mortality prediction in patients with COVID-19 pneumonia.

**Methods:**

A systematic search was conducted on Web of Science, PubMed, Embase, Scopus, and Google Scholar databases between December 2019 and September 2021. The meta-analysis was performed using the random-effects model, and sensitivity and specificity (with 95%CIs) of CT-SS were calculated using the study authors’ pre-specified threshold.

**Results:**

Sensitivity estimates ranged from 0.32 to 1.00, and the pooled estimate of sensitivity was 0.67 [95%CI (0.59–0.75)]. Specificity estimates ranged from 0.53 to 0.95 and the pooled estimate of specificity was 0.79 [95%CI (0.74–0.84)]. Results of meta-regression analysis showed that radiologist experiences did not affect the sensitivity and specificity of CT-SS to predict mortality in COVID-19 patients (*P* = 0.314 and 0.283, respectively). The test for subgroup differences suggests that study location significantly modifies sensitivity and specificity of CT-SS to predict mortality in COVID-19 patients. The area under the summary receiver operator characteristic (ROC) curve was 0.8248.

**Conclusion:**

Our results have shown that CT-SS has acceptable prognostic accuracy for mortality prediction in COVID-19 patients. This simple scoring method could help to improve the management of high-risk patients with COVID-19.

## Introduction

In December 2019, coronavirus disease 2019 (COVID-19) emerged from Wuhan, China [1, 2]. This novel coronavirus disease caused by severe acute respiratory syndrome coronavirus 2 (SARS-CoV-2) was highly transmissible. The basic reproductive ratio (R0) of SARS-CoV-2 ranges from 2.2 to 3.9 [3, 4]. Therefore, COVID-19 spread rapidly throughout the world and the World Health Organization (WHO) declared it a pandemic on March 11, 2020 [5]. From December 2019 to October 8, 2021, a total of 237,809,466 confirmed cases and 4,853,001 deaths due to COVID-19 had been reported across the world [6]. Due to the high mortality rate of COVID-19, identification of the potential prognostic factors associated with the fatal outcomes would play a critical role to predict different orders of risk for COVID-19 patients. Early identification of patients at higher risk of death would help to improve patient management and better allocation of medical resources.

Studies have focused on determining different prognostic factors such as laboratory tests, comorbidities, and radiological manifestations [7–13]. Computed tomography (CT) as the most common method used in the diagnosis of COVID-19 has a high sensitivity to depict pulmonary pneumonia [14]. Chest CT findings provide great added value in characterizing the extent of disease and severity of pulmonary involvements [15]. This approach reports the extent of pulmonary involvement in chest CT severity score (CT-SS), which would help in clinical decision-making for symptomatic patients or even those without clinical symptoms. In retrospective case series, investigators have shown that the extent of lung damage was more pronounced in deceased patients as compared to survivors [16]. Therefore, CT-SS could be considered as an appropriate prognostic factor for mortality prediction in patients with COVID-19 pneumonia.

However, while several case series have shown a significant correlation between CT-SS and mortality of the COVID-19 patients, there are no systematic review and meta-analysis reporting the prognostic accuracy of CT-SS for mortality prediction in COVID-19 patients. In this study, we performed a meta-analysis evaluating the prognostic accuracy of CT-SS for mortality prediction in patients with COVID-19 pneumonia.

## Methods

### Protocol of the systematic review and meta-analysis

This systematic review and meta-analysis was performed following a pre-defined protocol and reported in accordance with the preferred reporting items for systematic reviews and meta-analyses (PRISMA) checklist [17].

### Information sources and search strategies

Web of Science, PubMed, Embase, Scopus, and Google Scholar were searched to find studies reporting prognostic accuracy of CT-SS for mortality prediction in patients with COVID-19 pneumonia between December 2019 and September 2021. The review was conducted using the following keywords and logical operators: ((covid-19 OR SARS-CoV-2 OR 2019 Novel Coronavirus OR 2019-nCoV OR Wuhan virus OR severe acute respiratory syndrome coronavirus 2 OR coronavirus disease 2019) AND (CT OR computed tomography OR Chest computed tomography OR Chest CT OR X-Ray CT Scan OR X-Ray CAT Scan OR CT scan OR CAT scan) AND (Mortality OR Death OR decease* OR died OR dead)). The bibliographic lists of included articles were also reviewed.

Literature screening and assessment of the studies for inclusion were independently performed by two reviewers (NN and DR). Any disagreement was resolved by consulting a third investigator (SSZ).

The original studies investigating the prognostic performance of CT-SS for mortality prediction in COVID-19 patients were eligible to be included. Studies with unavailable full texts and insufficient data to calculate sensitivity and specificity were excluded.

### Study selection and data collection process

Two reviewers (NN and DR) independently extracted principal study characteristics from the included studies. The first author of the selected articles, publication year, country, mean age of patients, sensitivity, and specificity (with 95%CIs) of CT-SS for mortality prediction in COVID-19 patients, sample size, and the gender ratio of males were extracted. The extracted data were checked by the third author (SSZ), and any disagreement between the authors was resolved through discussion. The characteristics of included studies are presented in Table 1.

**Table 1:** Characteristics of included studies

Study	Publication year	Country	Sample size(N)	Age (mean ± SD)	Male (N)	Sensitivity	95%CI	Specificity	95%CI
Abdollahi et al. [18]	2021	Iran	742	56.59 ± 14.88	451 (60.8%)	0.63	0.54–0.71	0.53	0.48–0.58
Angeli et al. [15]	2021	Italy	301	69.8 ± 13.0	209 (69.4%)	0.38	0.29–0.47	0.92	0.87–0.96
Bayrak et al. [19]	2021	Turkey	86	71.1 ± 14.1	61 (70.9%)	0.42	0.28–0.58	0.77	0.56–0.91
Besutti et al. [20]	2021	Italy	866	59.8 (50.2–72.5)*	527 (60.85%)	0.57	0.46–0.67	0.83	0.80–0.85
Charpentier et al. [21]	2021	France	210	66 ± 16	146 (69.5%)	0.75	0.60–0.86	0.64	0.56–0.71
Dilek et al. [22]	2021	Turkey	100	61 ± 14.85	61 (61%)	0.76	0.62–0.87	0.66	0.51–0.79
Guillo et al. [23]	2020	France	214	59 ± 19	119 (56%)	0.68	0.49–0.83	0.74	0.64–0.82
Hajiahmadi et al. [24]	2021	Iran	192	57.5 ± 1.11	114(59.38%)	0.67	0.49–0.81	0.73	0.65–0.80
Isik et al. [25]	2021	Turkey	257	52 ± 14.62	142 (55.3%)	0.74	0.49–0.91	0.65	0.59–0.71
Kazemi et al. [26]	2020	Iran	91	58.04 ± 16.5	57 (62.6%)	0.67	0.51–0.81	0.69	0.54–0.81
Kimura-Sandoval et al. [27]	2021	Mexico	166	50 ± 14	100(60.2%)	0.83	0.66–0.93	0.82	0.74–0.88
Li et al. [28]	2021	China	147	66 (57–72)*	83 (54%)	0.50	0.29–0.71	0.90	0.84–0.95
Li et al. [29]	2020	China	102	57 (45–70)*	59 (58%)	0.64	0.31–0.89	0.95	0.76–1.00
Li et al. [30]	2020	China	83	45.5 ± 12.3	44 (53.0%)	0.80	0.59–0.93	0.83	0.71–0.91
Li et al. [31]	2020	China	46	71.1 ± 8.5	65 (66.3%)	0.83	0.63–0.95	0.77	0.55–0.92
Magdy et al. [32]	2021	China	266	34.75 ± 10.7	176(66.17%)	1.00	0.69–1.00	0.92	0.85–0.98
Mirza-Aghazadeh-Attari et al. [33]	2020	Iran	50	65.4 ± 16.77	27(54%)	0.74	0.52–0.90	0.63	0.42–0.81
Raoufi et al. [34]	2020	Iran	380	53.62 ± 16.66	251 (66.1%)	0.76	0.56–0.90	0.76	0.71–0.80
Ruch et al. [35]	2020	France	572	66.0 ± 16.0	343 (60.0%)	0.32	0.26–0.39	0.92	0.89–0.95
Salahshour et al. [36]	2020	Iran	739	49.2 ± 17.2	419(56.7%)	0.64	0.44–0.81	0.87	0.83–0.90
Salvatore et al. [37]	2021	Italy	103	61.0 (23.0–91.0)***	59 (60.20%)	0.53	0.27–0.79	0.84	0.70–0.93
Tabatabaei et al. [38]	2020	Iran	90	44.2 ± 5.9 and 44.3 ± 5.9 for non-survivors and survivors, respectively	54(60%)	0.83	0.65–0.94	0.87	0.75–0.94
Yuan et al. [13]	2020	China	27	60 (47–69)*	12(44.44%)	0.86	0.42–1.00	0.82	0.57–0.96
Zhou et al. [39]	2020	China	134	48 (38–61)* and 68 (59–76)* for survivors and non-survivors, respectively	85(63.43%)	0.69	0.57–0.80	0.82	0.70–0.91

*Median (IQR), ***median (range)

### Summary measures and synthesis of results

Data synthesis was conducted using Stata version 11.0 (Stata Corporation, College Station, TX, USA) and Review Manager 5.3 software (The Nordic Cochrane Centre, The Cochrane Collaboration). For each study, sensitivity and specificity (with 95% CIs) of CT-SS for mortality prediction in COVID-19 patients were calculated using the study authors’ pre-specified threshold.

Heterogeneity between studies was assessed using Cochran’s Q test and the Inconsistency index (I2) test (heterogeneity was defined as *P* < 0.1). For further assessment of the possible sources of between-study heterogeneity, the meta-regression and subgroup analysis were applied. The test for subgroup differences was performed according to study locations to evaluate whether study locations affects the sensitivity and specificity of CT-SS to predict mortality in COVID-19 patients. For meta-regression analysis, the moderator variable was the radiologist experience and it was plotted against sensitivity and specificity of CT-SS. Visual inspection of the generated funnel plot was employed to evaluate publication bias among studies. The summary ROC curve was also generated to evaluate the overall prognostic performance of CT-SS for mortality prediction in COVID-19 patients.

## Results

### Study selection

**Fig. 1:**
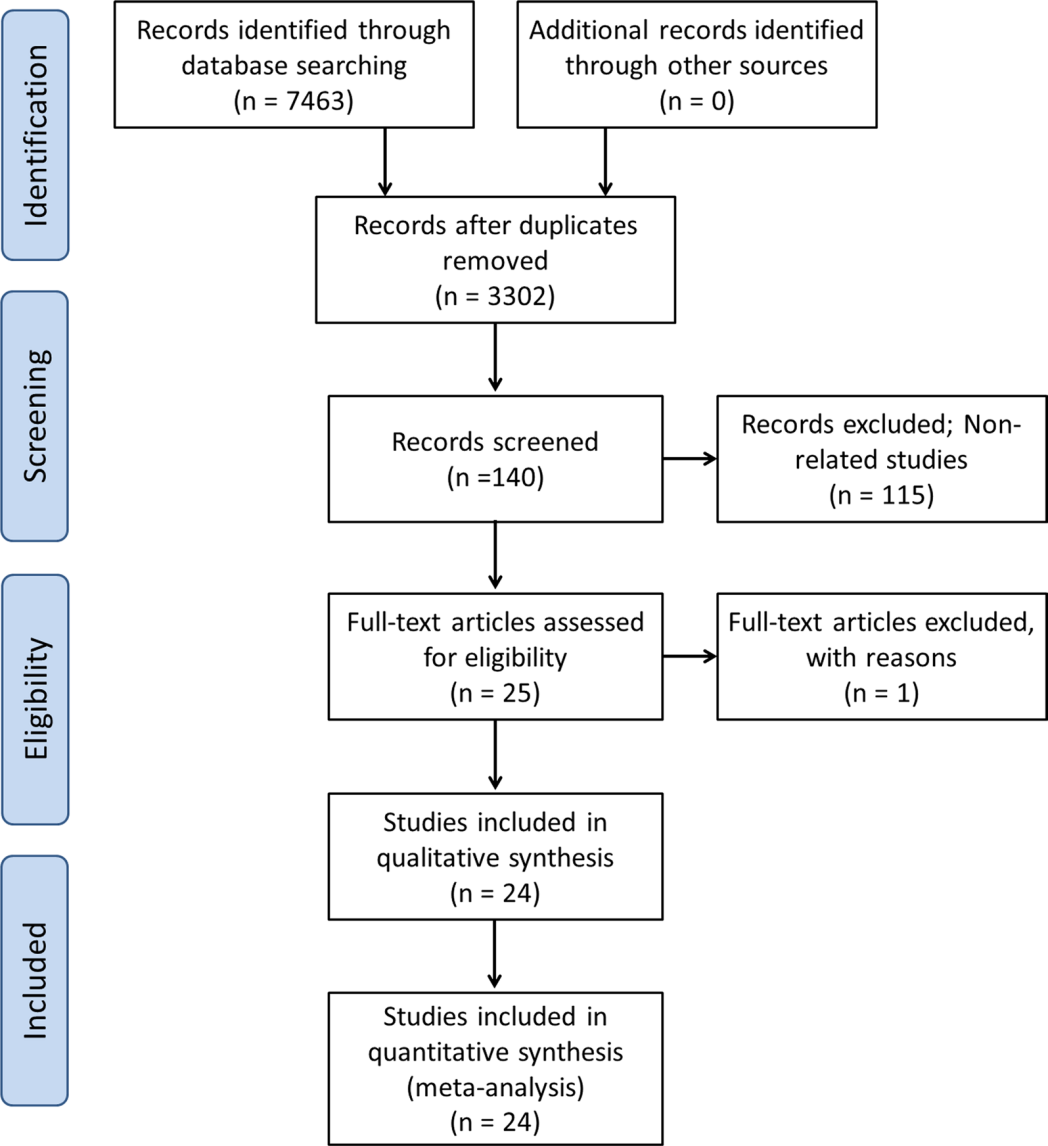
PRISMA flow diagram details the literature search process

The literature search process is detailed in Fig. 1. The search strategy retrieved 7463 studies. Of the 3302 records after removing duplicate records, 25 articles were selected for further assessment based on the title and abstract. After the full-text assessment of the remaining studies, one study was excluded because it did not provide sufficient data to calculate sensitivity and specificity. Twenty-four studies were eventually included in the meta-analytical processes.

### Study characteristics

The characteristics of the included studies are presented in Table 1. The first author, publication year, country, mean age of patients, sample size, and the gender ratio of males have been presented in the table. For each study, the sensitivity and specificity (with 95%CIs) of CT-SS were also listed in the table.

### Risk of bias within studies

The *P*-values obtained from the Chi-squared test of heterogeneity were < 0.001 for sensitivity and specificity of CT-SS. Moreover, the results of *I*^2^ test for sensitivity and specificity of CT-SS were calculated as 87.8% and 92.5%, respectively. Therefore, the random-effects model of the meta-analysis was applied for evaluating the prognostic accuracy of CT-SS for mortality prediction in patients with COVID-19 pneumonia.

### Synthesis of results

**Fig. 2:**
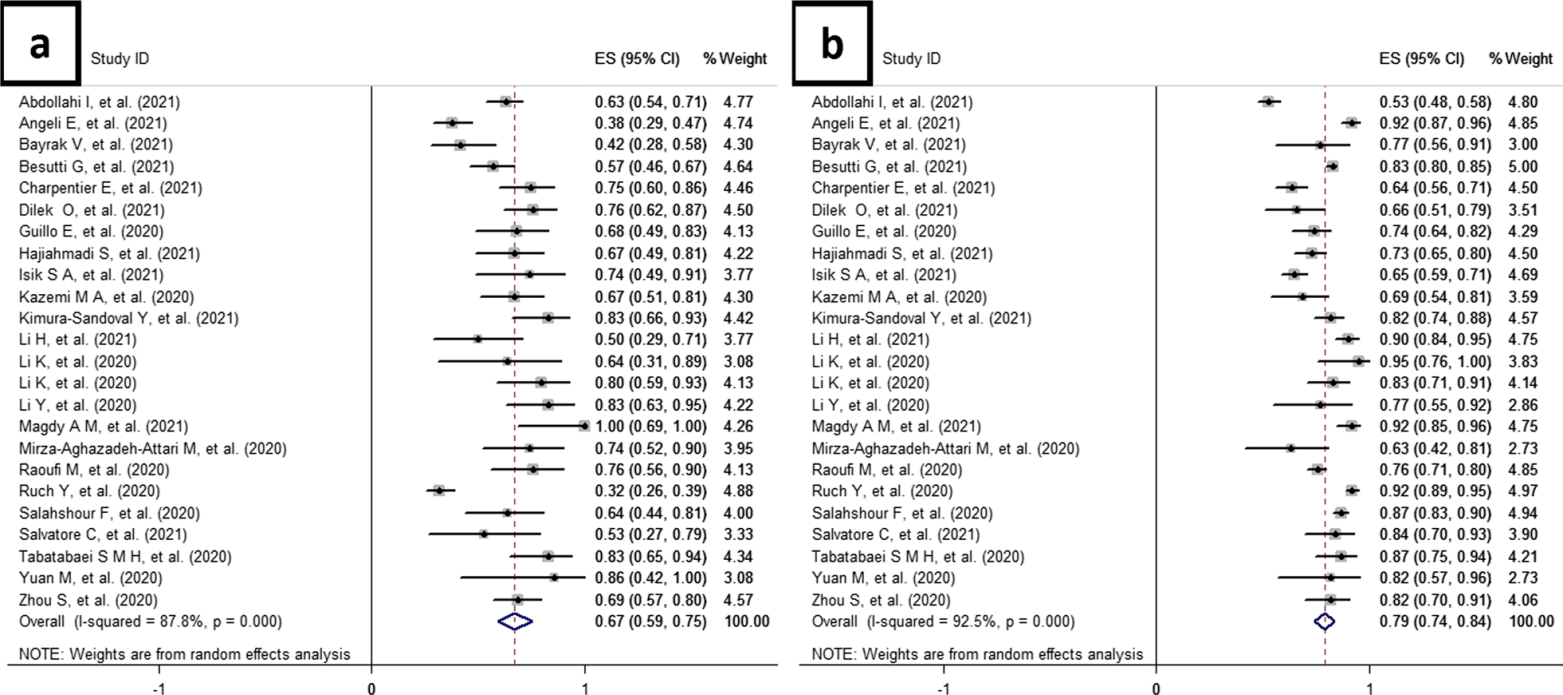
Forest plot for 24 included studies. **a** In this plot, pooled data evaluating the sensitivity of CT-SS to predict mortality in COVID-19 patients have been demonstrated under the random-effects model. The pooled estimate for sensitivity was calculated as 0.67 (95% CI, 0.59–0.75). **b** In this plot, pooled data evaluating the specificity of CT-SS to predict mortality in COVID-19 patients have been demonstrated under the random-effects model. The pooled estimate for specificity was calculated as 0.79 (95% CI, 0.74–0.84)

The forest plots of sensitivities and specificities of CT-SS for mortality prediction in COVID-19 patients are presented in Fig. 2a, b, respectively. Sensitivity estimates ranged from 0.32 to 1.00, and the pooled estimate of sensitivity was 0.67 [95% CI (0.59–0.75)] (Fig. 2a). Specificity estimates ranged from 0.53 to 0.95, and the pooled estimate of specificity was 0.79 [95% CI (0.74–0.84)] (Fig. 2b).

### Risk of bias across studies

**Fig. 3:**
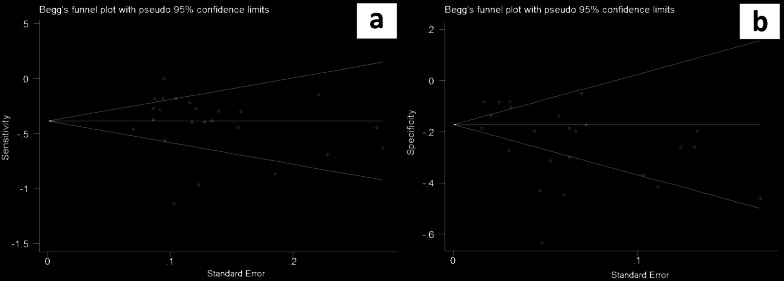
Funnel plots for 24 included studies. Visual inspection of the generated funnel plots was employed to evaluate publication bias among studies. The funnel plots appear asymmetrical. **a** In this plot, the X and Y axes represent sensitivity and standard errors, respectively. **b** In this plot, the X and Y axes represent specificity and standard errors, respectively

In Fig. 3, the funnel plots were considered to be moderately asymmetrical in shape which demonstrates the existence of publication bias in the results of included studies.

The *P*-values obtained in meta-regression analysis to evaluate the effect of the radiologist experiences on the sensitivity and specificity of CT-SS were *P* = 0.314 and 0.283, respectively (analysis not presented).

**Fig. 4:**
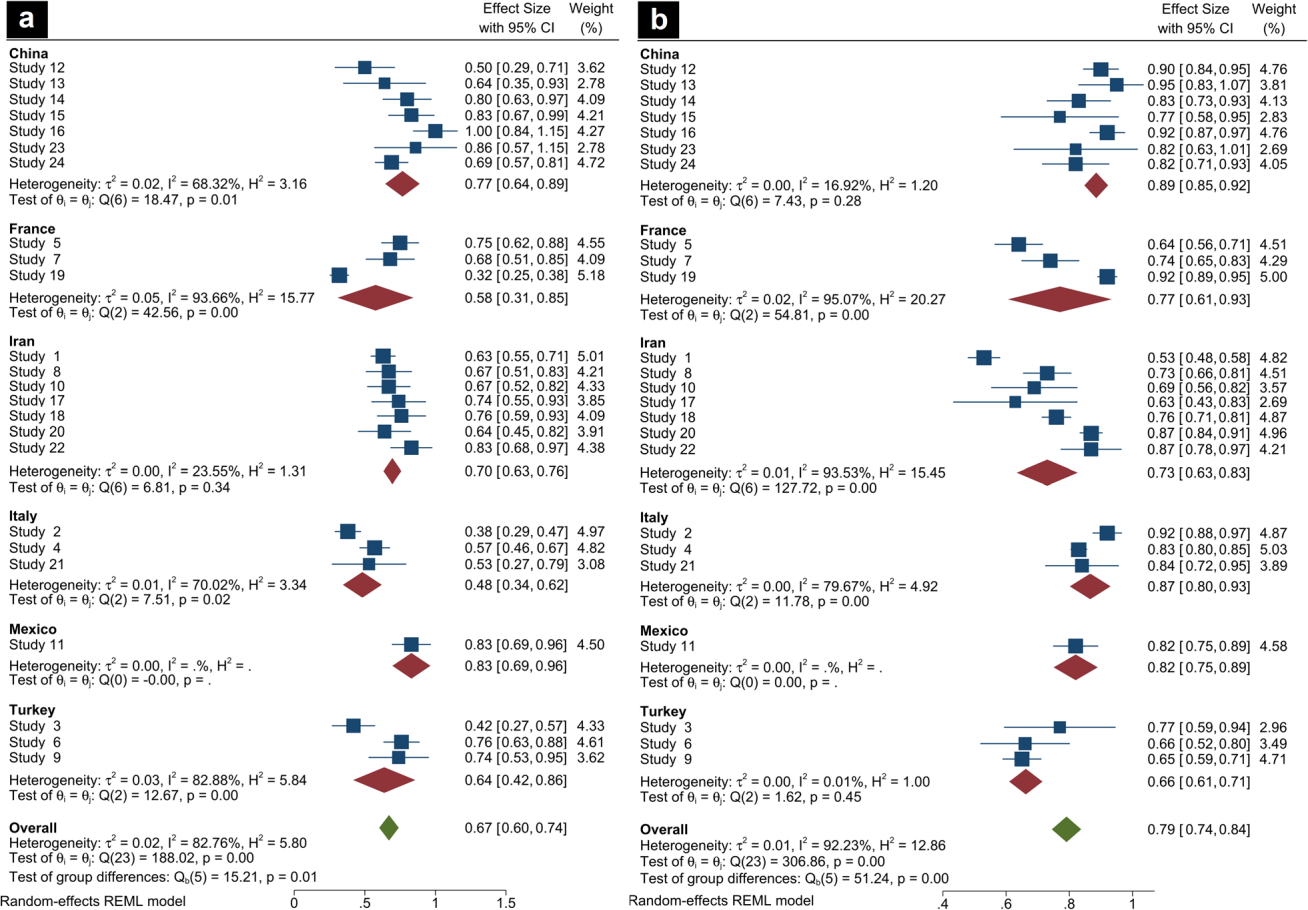
Forest plot for 24 included studies according to the location of studies. **a** In this presentation, pooled data evaluating the sensitivity of CT-SS to predict mortality in COVID-19 patients have been demonstrated for each country under the random-effects model. **b** In this presentation, pooled data evaluating the specificity of CT-SS to predict mortality in COVID-19 patients have been demonstrated for each country under the random-effects model

The sensitivity and specificity of CT-SS for mortality prediction of COVID-19 patients were categorized and subgrouped according to study locations. The overall effect size for each subgroup was calculated and is shown in Fig. 4.

**Fig. 5:**
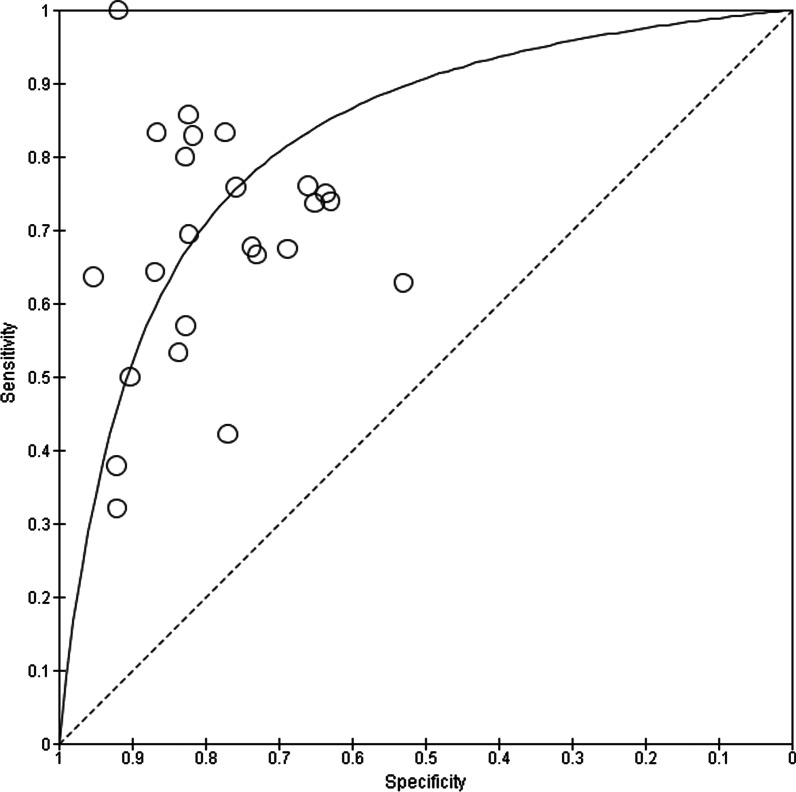
Summary ROC plot for included studies. Black line denotes summary ROC curve and circles represent data points

The corresponding summary ROC plot is shown in Fig. 5. The area under the summary ROC curve was 0.8248.

## Discussion

Severe or critical patients with COVID-19 are of great concern. They mostly have a poor prognosis and high mortality [40, 41]. Effective predictive models for early identification of patients at higher risk of death would improve patient management and help clinicians decide what intensity of care each patient needs. In the last two years, chest imaging combined with clinical evaluation and laboratory tests had played an essential role in patient management. In addition to symptomatic patients, radiographic lung injury abnormalities could even be manifested in asymptomatic cases. In Xie et al. study, chest CT findings of patients with COVID-19 infection who had initial negative reverse-transcriptase polymerase chain reaction results were reported. Repeated swab tests eventually confirmed COVID-19 infection of the patients [4]. The diagnostic value of radiological manifestations is already recognized for COVID-19.

Although CT is now a common method in the diagnosis of COVID-19, there is a lack of clinical evidence about its prognostic role for mortality prediction in COVID-19 patients. Deceased patients had higher CT-SSs versus those who recovered [16] which suggests that severe radiological manifestations may indicate a poor prognosis. In this study, we investigated the prognostic accuracy of CT-SS for mortality prediction in COVID-19 patients by conducting a meta-analysis. To date, no meta-analysis studies have been conducted on this aspect. To our knowledge, this report is the first meta-analysis describing the prognostic accuracy of CT-SS for mortality prediction in COVID-19 patients.

The retrieved studies have investigated deceased and survival patients with COVID-19 pneumonia, which allows an assessment of the overall CT performance in terms of sensitivity and specificity. All studies are retrospective and researchers had known the outcomes of patients. There is no potential risk of bias regarding the index test because a pre-specified diagnostic threshold was used for CT interpretations.

The results of our meta-analysis showed that CT-SS has, respectively, achieved pooled sensitivity and specificity values of 67% (95% CI, 59–75%) and 79% (95% CI, 74– 84%) in mortality prediction of COVID-19 patients. The classifiers have inherent strengths and limitations. There are two reasons to observe false negative results in radiological manifestations of COVID-19 patients. First, symptomatic patients may not have pulmonary involvement in the early course of the disease [41, 42]. Second, pneumonia may not develop in symptomatic upper respiratory tract infections [43, 44]. On the other hand, patient infection with other viral types of pneumonia such as various forms of flu could also result in false positive cases [45].

Radiologist experience is one of the effective factors which may have affected the predictive performance of CT-SS [46]. Results of meta-regression analysis showed that radiologist experiences did not affect the sensitivity and specificity of CT-SS to predict mortality in COVID-19 patients (*P* = 0.314 and 0.283, respectively). The test for subgroup differences based on study location suggests that there is a statistically significant subgroup effect for sensitivity and specificity of CT-SS (*P* = 0.01 and < 0.01, respectively), meaning that study location significantly modifies sensitivity and specificity of CT-SS to predict mortality in COVID-19 patients. These differences can be due to the heterogeneity in the severity of illness and epidemic [46]. There are also differences in the experience of treatment staff, health care, and hospital equipment in studied countries which may cause different mortality rates.

In summary, in ROC curve analysis, a considerable area under the curve was achieved for CT-SS. Therefore, CT-SS has acceptable performance for mortality prediction in COVID-19 patients. A simple and rapid approach with high sensitivity, which results in a low number of false negatives, is the preferred method for mortality prediction of COVID-19 patients. Such a system could help to improve the management of high-risk patients with COVID-19 even when they are clinically silent. From the results, it could be concluded that CT-SS has acceptable prognostic accuracy for mortality prediction in COVID-19 patients. This simple scoring method could help for triage of patients and screening of the patients with a higher need for intensive care.

Despite the significant prognostic value of the CT-SS, some limitations should be acknowledged for this parameter. There is a wide scoring range from 20 to 40 regions in the reported scores which makes assessments more difficult. Therefore, this parameter is inherently complex and time-consuming for clinical use. Second, the right lung is larger than the left one. So, their corresponding lobes and segments have different sizes. Dedicated software and an experienced specialist are required to consider these differences in semiquantitative and quantitative studies [47].

## Conclusion

In this study, the prognostic accuracy of CT-SS for mortality prediction in COVID-19 patients was investigated. Our results have shown that CT-SS has acceptable prognostic accuracy for mortality prediction in COVID-19 patients. This simple scoring method could help to improve the management of high-risk patients with COVID-19.

## Data Availability

All data generated or analyzed during this study are included in this published article.

## Abbreviations

COVID-19: Coronavirus disease 2019
SARS-CoV-2: Severe acute respiratory syndrome coronavirus 2
CT-SS: Chest CT severity score
CT: Computed tomography
CI: Confidence interval
Summary ROC: Summary receiver operator characteristic curve
